# Supplementary material for: Low Doses of Silver Nanoparticles Selectively Induce Lipid Peroxidation and Proteotoxic Stress in Mesenchymal Subtypes of Triple-Negative Breast Cancer
Source: Cancers (Basel). 2021 Aug 22;13(16):4217. doi: 10.3390/cancers13164217 (PMC8393662; doi:10.3390/cancers13164217)
Supplement: Supplementary file 1 [file cancers-13-04217-s001.zip › cancers-1283072_supplementary.pdf]

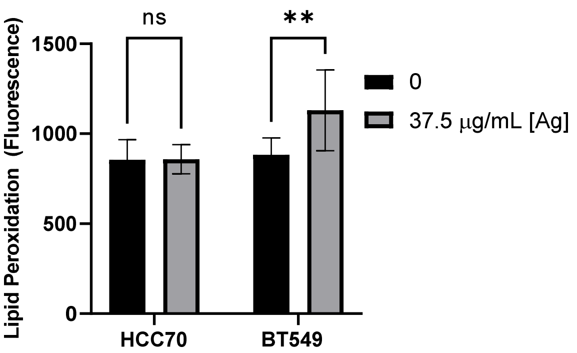

Figure S1. *AgNPs induce lipid peroxidation in CLBC but not BLBC.* To assess lipid peroxidation, cells were treated with AgNPs for 24 hours, stained with Liperfluo, and fluorescence was assessed by flow cytometry. Data were obtained from 3 biological replicates per condition and 3 independent experiments. Statistical analysis was performed by two-way ANOVA followed by post-hoc Tukey test. Significant differences are indicated (**$p<0.01$).

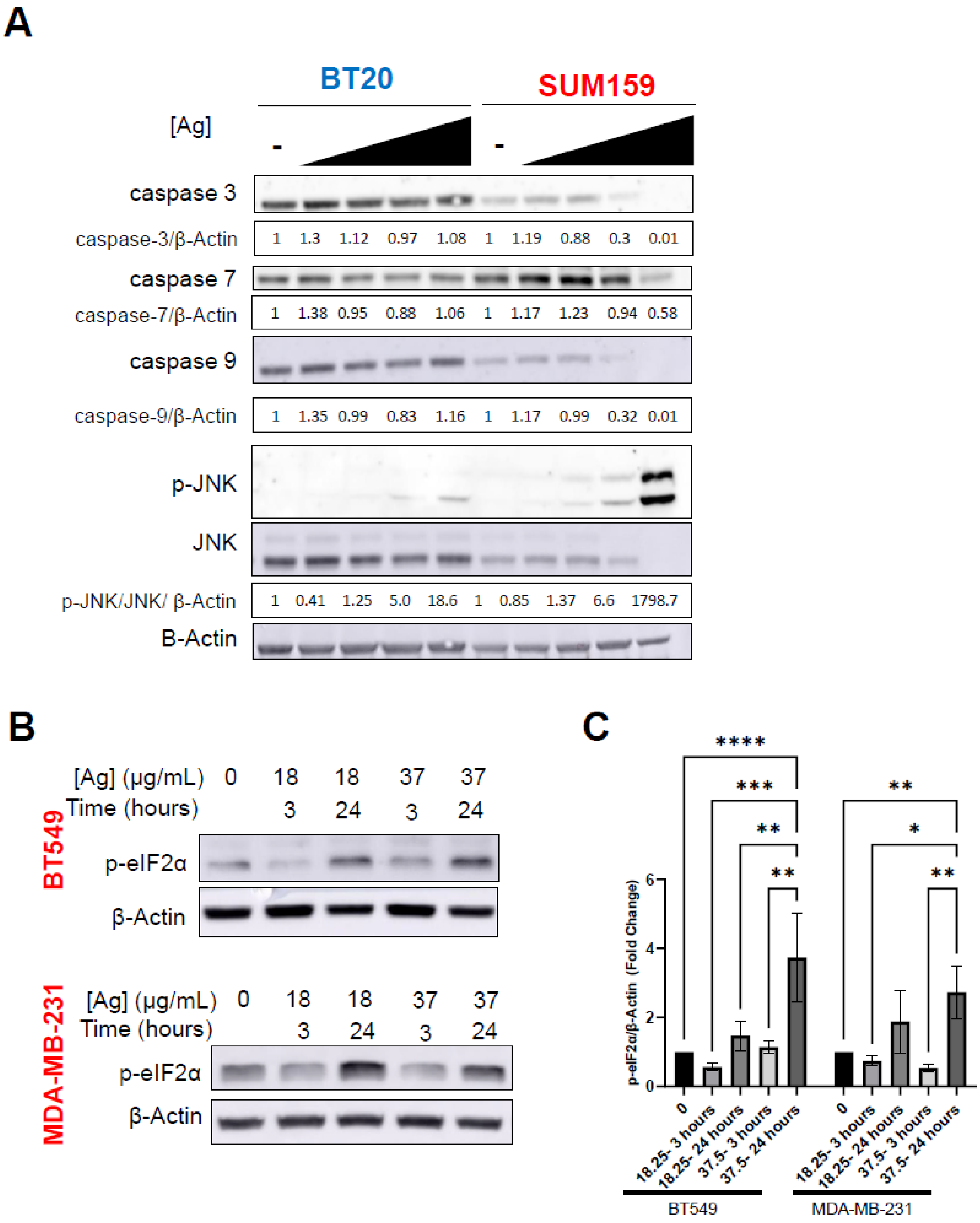

Figure S2. *AgNPs induce time dependent activation of proteotoxic stress responses in CLBC cells.* **(A)** BLBC (blue) and CLBC (red) cells were treated with (left to right) 0, 9.375, 18.75, 37.5, or 75 μg/mL AgNPs for 24 hours and protein expression was evaluated by western blot. **(B)** CLBC cells (red) were treated with 0, 18.25, or 37.5 μg/mL AgNPs for 3 or 24 hours and protein expression was evaluated by western blot. **(C)** Densitometry was performed on at least three independent experiments per cell line and the ratio of p-eIF2α to β-actin is shown. Statistical analysis was performed by two-way ANOVA followed by post-hoc Tukey test. Significant differences are indicated (****p<0.0001, ***p<0.001, **p<0.01, *p<0.05).
