# Supplementary material for: Low Doses of Silver Nanoparticles Selectively Induce Lipid Peroxidation and Proteotoxic Stress in Mesenchymal Subtypes of Triple-Negative Breast Cancer
Source: Cancers (Basel). 2021 Aug 22;13(16):4217. doi: 10.3390/cancers13164217 (PMC8393662; doi:10.3390/cancers13164217)

Figure 4: Uncropped Western Blots

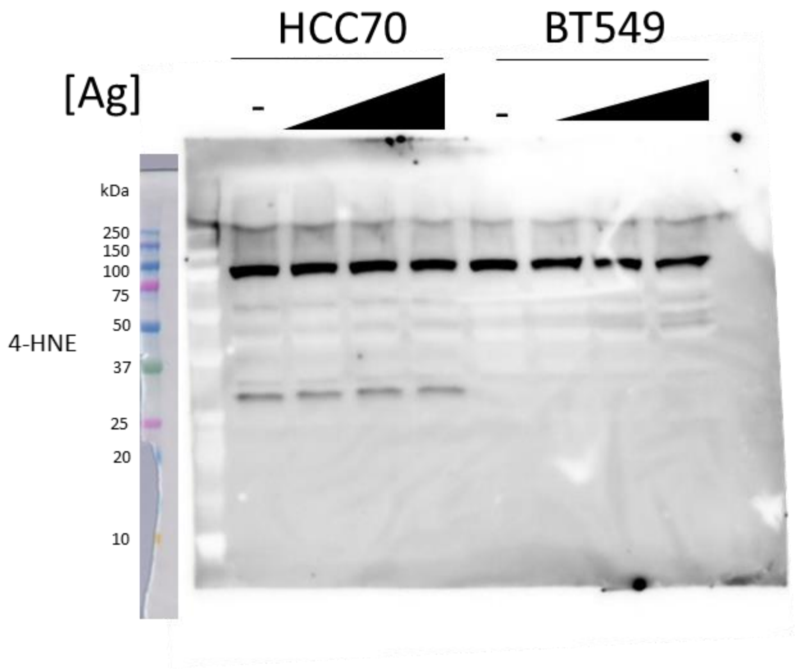

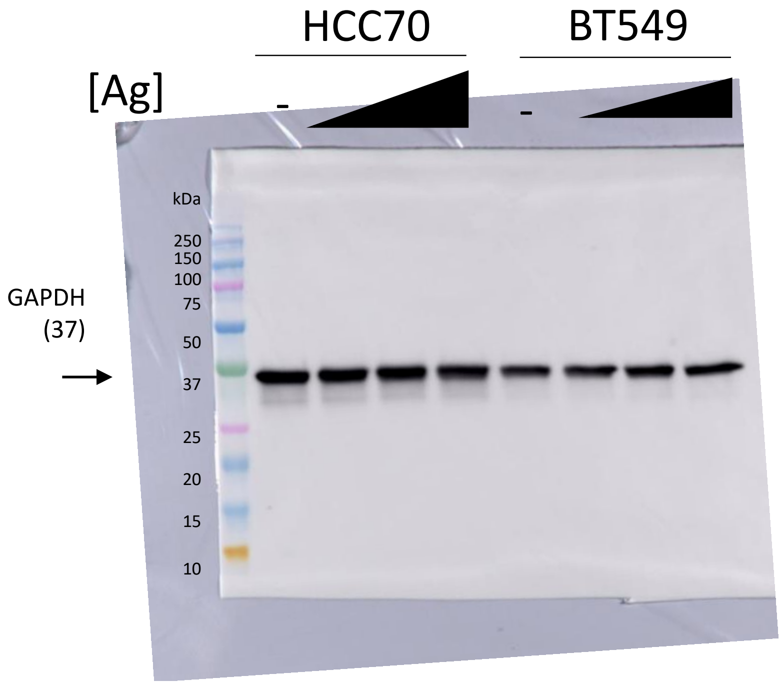

Figure 5 Uncropped Western Blots- Part 1

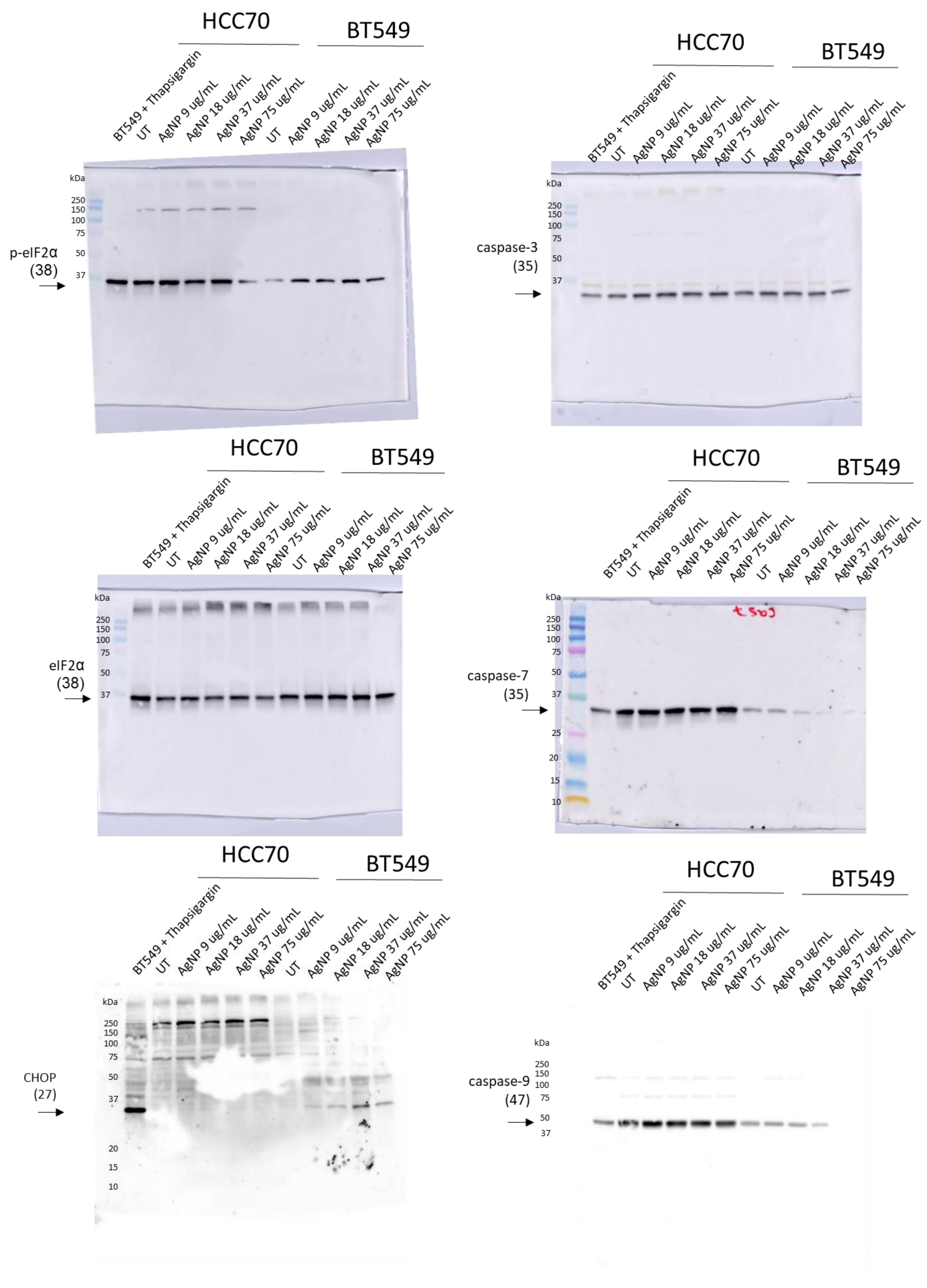

Figure 5 Uncropped Western Blots- Part 2

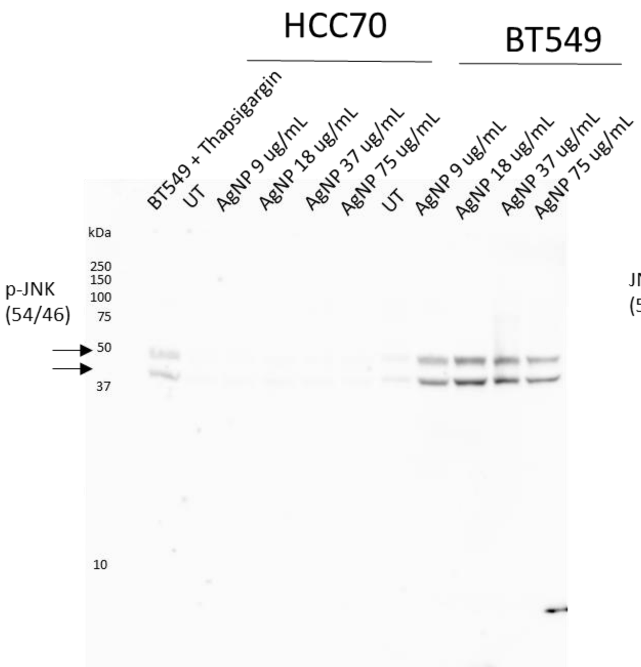

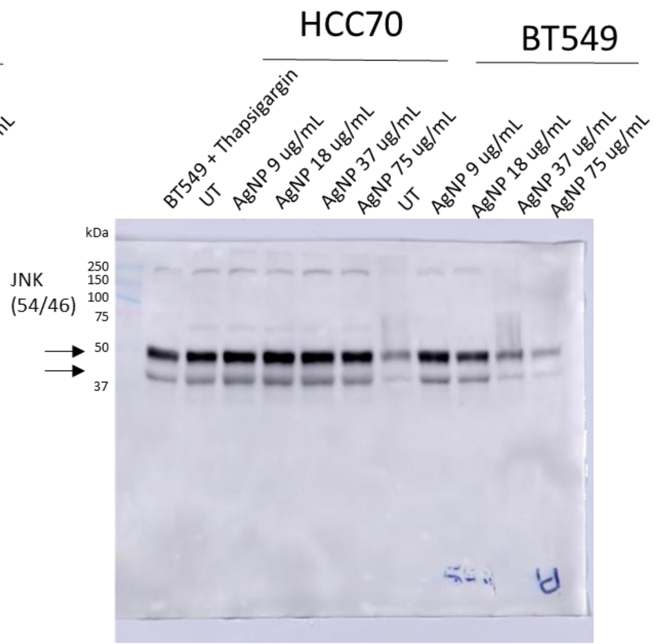

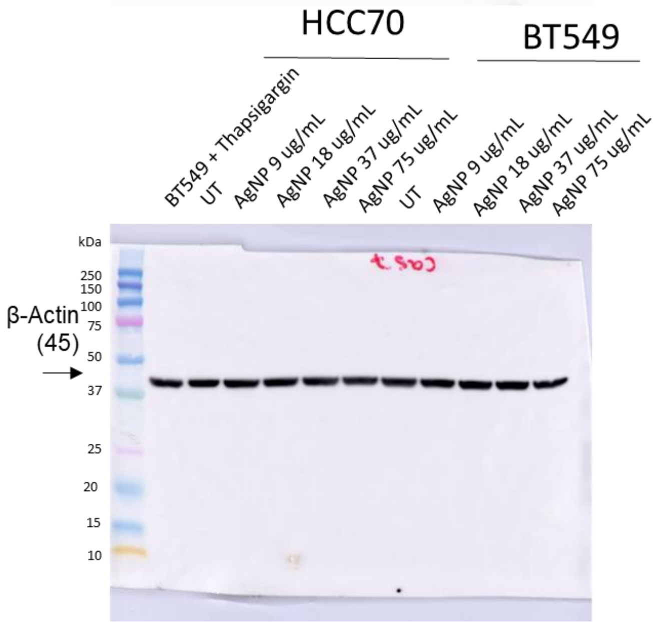

Supplementary Figure S2: Uncropped Western Blots Part 1

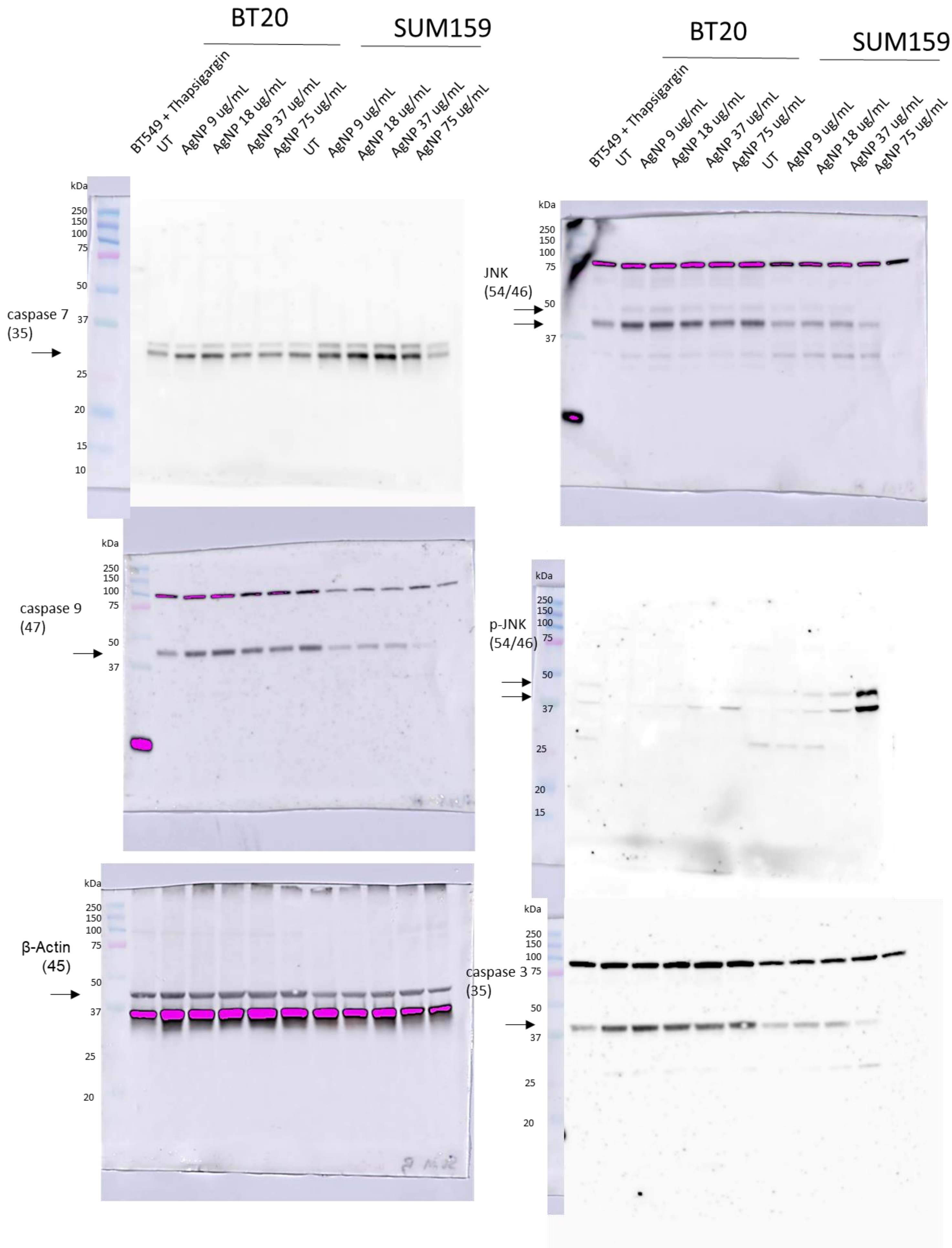

Supplementary Figure S2: Uncropped Western Blots Part 2

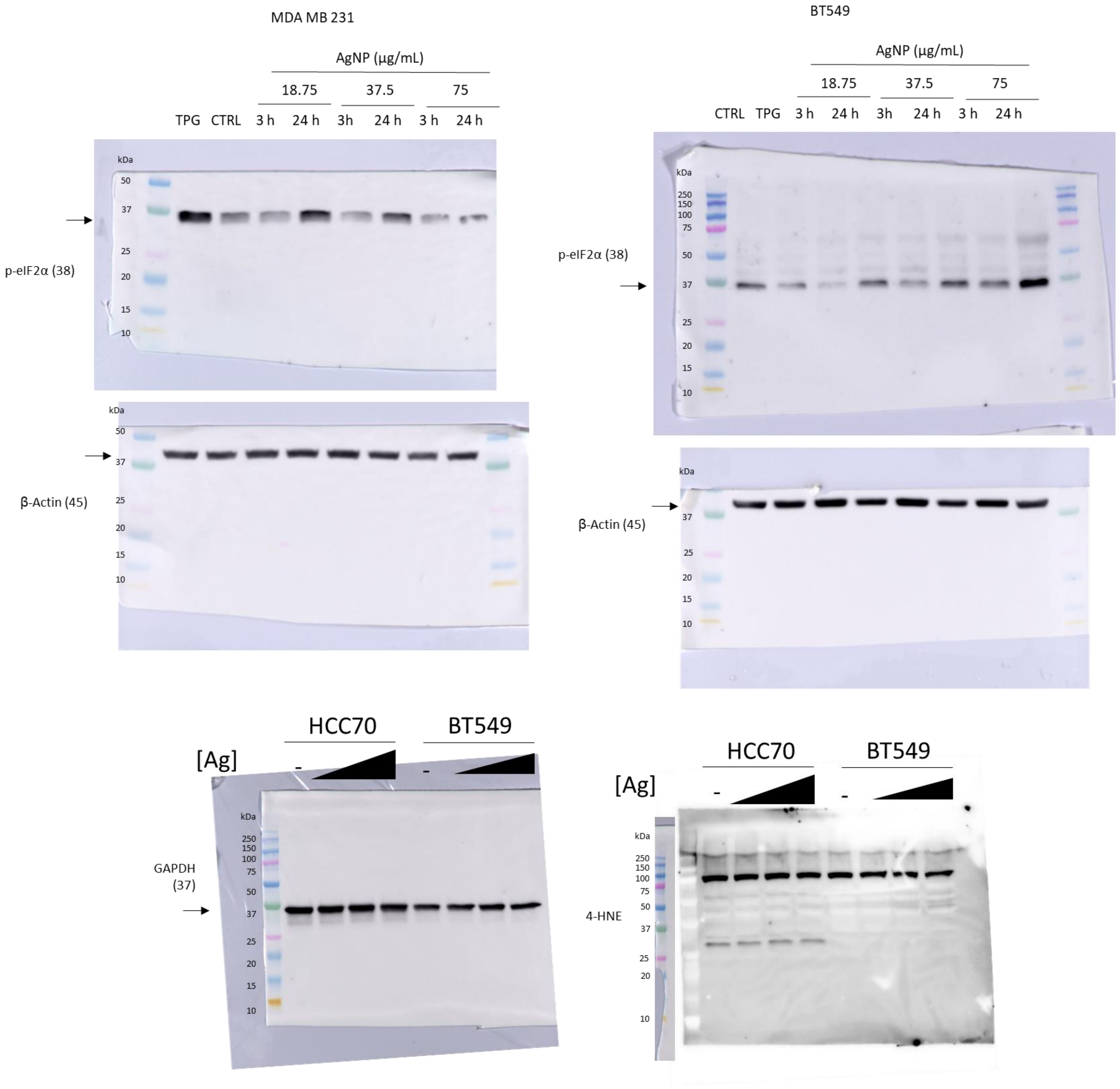

Supplement: Supplementary file 1 [file cancers-13-04217-s001.zip › cancers-1283072_western blot.pdf]
